# Implementation and experience of an innovative smart patient care system: a cross-sectional study

**DOI:** 10.1186/s12913-022-07511-7

**Published:** 2022-01-29

**Authors:** Ming-Huan Wen, Dorothy Bai, Shirling Lin, Chi-Jen Chu, Yeh-Liang Hsu

**Affiliations:** 1 Department of Nursing, Taipei Veterans General Hospital, Taipei, Taiwan; 2 School of Nursing, National Yang Ming Chiao Tung University, Taipei, Taiwan; 3 Gerontechnology Research Center, Yuan Ze University, Taoyuan, Taiwan; 4 Department of Medicine, Taipei Veterans General Hospital, Taipei, Taiwan; 5 Mechanical Engineering Department, Yuan Ze University, Taoyuan, Taiwan

**Keywords:** Nurse call system, Patient care system, Implementation, Patient communication, False alarm, Bed exit

## Abstract

**Background:**

Although a patient care system may help nurses handle patients’ requests or provide timely assistance to those in need, there are a number of barriers faced by nurses in handling alarms.

**Methods:**

The aim of the study was to describe the implementation and experience of an innovative smart patient care system (SPCS). This study applied a cross-sectional descriptive design. We recruited 82 nurses from a medical center in Taiwan, with 25 nurses from a ward that had introduced an SPCS and 57 nurses from wards that used the traditional patient care system (TPCS). The major advantages of the SPCS compared to the TPCS include the specification of alarm purposes, the routing of alarms directly to the mobile phone; the capability of immediate communication via phone; and three-stage bed-exit alerts with low false alarm rate.

**Results:**

Approximately 56% of nurses in the TPCS wards perceived that the bed-exit alert was easily ignorable, while this rate was reduced to 32% in the SPCS ward. The immediate communication via phone was considered as the most helpful function of the SPCS, with a weighted average score of 3.92/5, and 52% of nurses strongly agreed (5/5) that this function was helpful. The second-highest ranked function was the three-stage bed-exit alert, with an average score of 3.68/5, with approximately 24% of nurses strongly agreeing (5/5) that this function was helpful. The average response time using TPCS was 145.66 s while it was 59.02 s using the SPCS (*P* < .001). Among the 110 observed alarms in the SPCS ward, none of them were false bed-exit alarms. In comparison, among 120 observed alarms in the TPCS wards, 42 (35%) of them were false bed-exit alarms (*P* < .001). In this study, we found that 30.91% of alarms using SPCS were processed because nurses received and responded to the alert via mobile phone.

**Conclusions:**

A smart patient care system is needed to help nurses make more informed prioritization decisions between responding to alarms and ongoing tasks and finally assist them in adjusting their work in various situations to improve work efficiency and care quality.

## Introduction

Patient care systems have been commonly used in hospitals. ‘A system of call bell’, described by Florence Nightingale in the mid-nineteenth century, may be one of the earliest patient care or nurse call system concepts [1]. Although the forms of the patient care system vary from a drawstring-bell system to a computer-based system, the core function of notifying nurses that a patient needs their assistance remains. Existing findings show that the patient care system helps patients feel safe, increases their control of the situation, and achieves successful recovery [2–4]. Information transferred by the patient care system has been found to allow for timely information transfer, increase the timeliness of patient care, enhance problem-solving abilities, and facilitate teamwork among nurses [5].

In addition, the patient care system plays an important role in improving patient safety from the perspective of both nurses [6] and patients [7]. For example, patient falls are a significant issue in clinical settings [8–10]. The Centers for Medicare and Medicaid Services in the US have stopped paying for preventable hospital falls in 2008, which has raised more awareness of and efforts toward fall prevention in hospitals [11, 12]. Falls commonly occur between 5 pm and 7 am in a patient’s room, when staffing levels are lower, and a large number of falls are related to getting in and out of bed [13]. The modern patient care system often consists of a bed-exit alert that is automatically launched when a patient at risk of falling leaves the bed so that nurses can provide assistance when necessary.

Although the patient care system may help nurses handle patients’ requests or provide timely assistance to those in need, there are a number of barriers faced by nurses in handling alarms. Nurses have multiple clinical and administrative tasks, and the patient care system, by nature, is an interruption of ongoing tasks [14–16]. In human-computer interactions, interruptions have been found to negatively affect human cognition [17], which may negatively influence patient safety. Evidence has shown the relationships between interruptions and medical errors [17–19]. Conversely, not all interruptions caused by alarms are unwanted, and some are even desired to provide quality care [14, 20]. Among all the possible reasons behind alarms, such as information requirements, pain management, and toilet assistance, it has been shown that less than one-third of all nurse calls are considered serious or urgent [4, 6, 21]. Given that nurses have multiple tasks to handle, it is difficult and stressful for nurses to make decisions regarding whether or not to abort an ongoing task to handle an alarm [14].

With limited information on an alarm provided by patient care systems, nurses tend to lower their prioritization in responding to it [14, 22]. There are some other barriers, such as limited access to an alarm if it is only displayed in fixed places, such as monitors in the corridor or at nursing stations [23], a lack of ways to share alarm-responding responsibilities with colleagues [24], and insufficient time to respond to a bed-exit alert [25]. Responding to the demands of the current situation implies dealing with reality, which is an organizational ability [26]. To achieve this goal, a resilient patient care system is needed to help nurses make better decisions in various conditions and cope with the interruption to their work brought about by responding to an alarm. To date, limited evidence has been reported on resilient patient care systems designed with adaptive capacity that help nurses handle alarms [14].

The study aimed to describe the implementation and experience of an innovative smart patient care system (SPCS). The primary objectives were to compare the perception of the alarm handling process, response time, false alarm rates between SPCS and a traditional patient care system (TPCS) and to describe nurses’ user experience of SPCS.

## Methods

### Aim, design and setting

This study aimed to describe the implementation and experience of an innovative smart patient care system. In SPCS, all alarms can be routed to nurses’ mobile phones, allowing for immediate communication and three-stage bed-exit alerts. This study applied a cross-sectional descriptive design. We recruited nurses from four wards of the Gastroenterology and Hepatology Department of a medical center in Taiwan in 2018. It was a convenient sample as this department initiated a purchase of the smart system due to an increasing fall rates and the high false alarm rates of the traditional system, providing a good opportunity for us to explore the user experience of an innovative patient care system. The inclusion criteria were nurses who worked in the Gastroenterology and Hepatology Department, were over 20 years old, and spoke Chinese or Taiwanese. The exclusion criteria were nurses with working experience in the present department for less than 3 months. Nurses from four wards were recruited.

### Instruments

**Fig. 1:**
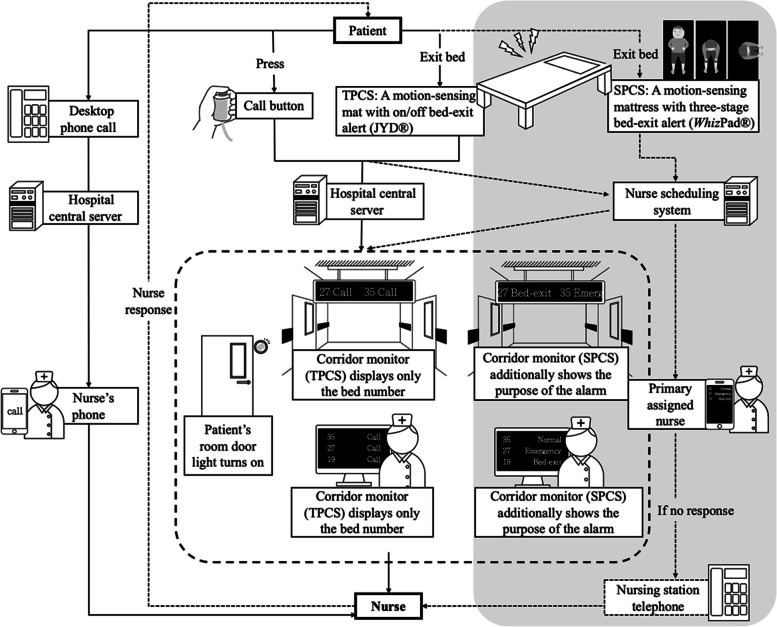
Comparison of the alarm signal pathway between the SPCS and the TPCS (the additional functions of the SPCS are in the gray area)

Among the four wards of the Gastroenterology and Hepatology Department, one ward had adopted an SPCS, while the other three used the TPCS. The TPCS had been the patient care system used in the study site for approximately two decades. The smart patient care system was introduced into one ward of the Gastroenterology and Hepatology Department in 2017, and all nurses in that ward were thereafter trained to use this system. A comparison between the SPCS and TPCS is presented in Fig. 1. In general, both the TPCS and SPCS are systems that nurses use to receive and handle nurse calls or bed-exit alerts. When patients press the call button, their room door light turns on, and their bed number is shown simultaneously on monitors in the corridor and nursing station, with an alarm sound raised. The display methods of bed-exit alerts are the same as those of a normal call in the TPCS. In the SPCS ward, all monitors in the corridor or nursing station not only show the bed number but also the purpose of the alert, such as normal, emergency, bed-exit, and desktop phone call, to assist nurses in prioritizing and arranging actions. Patients can press whichever button for proper assistance according to their situations. For example, they can press the emergency call button to call for the nurses’ immediate assistance if they decide it is an urgent situation.

A bed-exit alert is used for patients at risk of falling. In the TPCS wards, a movable sensing mat (JYD®) is used for bed-exit alerts, which are shown in the same way as a normal nurse call, without displaying the purpose as bed-exit. The bed-exit alert of this movable mat is only triggered when a patient leaves the mat. In comparison, the SPCS incorporates a motion-sensing mattress (*Whiz*Pad®) that applies a machine learning method that can identify users’ real-time positions, including on-bed, bed-edge, and off-bed positions. With these three-stage alerts, nurses can be notified when a patient initiates a departure from his/her bed as early as when he/she begins to sit up in bed. Furthermore, in the SPCS ward, the bed-exit alert can be stopped at the patient’s bedside, while in the TPCS wards, this alarm can be stopped remotely at nursing stations.

In addition, all SPCS alarms are additionally routed to nurses’ mobile phones so that they can receive all messages immediately when they are on duty. The information exchange system in the SPCS is also connected to the scheduling system in the hospital so that only nurses on duty and primarily assigned to a certain patient receive such alarms. When a nurse receives such an alarm, he/she can directly speak to patients when necessary via phone, and patients hear him/her through the speaker near their call button/bed.

### Study procedure

In terms of attitudes toward alarms, all the nurses from both the TPCS and SPCS wards were asked to choose the most annoying, cumbersome, time-consuming, and easily ignorable alarm. Nurses who worked in the SPCS ward were asked about their attitude toward and experience of how this system has helped them handle patient calls after using the system for 3 months or more. We developed a questionnaire for the user experience assessment since we failed to find an appropriate one in the existing literature. The questionnaire was then finalized after collecting experts’ opinions. The items included in the questionnaire could be divided into two categories. The first part was asking nurses to evaluate the specific functions of SPCS including the immediate communication via phone, three-stage bed-exit alert, event presentation, and the interface. The other part of items included care efficiency, the awareness of patients’ in-time situation, care quality, and working stress. We also asked nurses to specify their level of agreement or disagreement with the user experience statement using a 5-point Likert scale (strongly disagree, disagree, neutral, agree, and strongly agree) [27]. To compare nurses’ agreement levels for different statements, a weighted arithmetic mean was calculated. In addition to the subjective attitudes and using experiences, we observed the frequency of false alarms and the response time to alarms by a trained research nurse. Each nurse was observed two to three times on their responses to the alarms. The research nurse tracked the exact time of an alarm from ringing to being handled with a timer. A total of 230 alarms were observed with 120 alarms from the TPCS wards and 110 alarms from the SPCS ward. For each alarm, the research nurse recorded the alarm type, bed number, the responsible nurse, and the handling time for further analysis.

### Analysis

A descriptive analysis was conducted to describe the characteristics of participants using the mean, frequency, and percentage depending on the variables. For the linear variables including age, total working experience as a nurse, and working experience in the current department, t-tests were applied to compare the mean between TPCS and SPCS groups. For the comparison of sex between two groups, a Chi-square test was conducted. As for the other categorical variables including education level, job titles, and clinical nursing ladder, we used Fisher’s exact tests as one of the cells contained less than five cases. The clinical nursing ladder was categorized as N (less than 1 year of clinical experience and has not obtained any level of clinical nursing certificate), N1, N2, N3, and N4, according to the clinical nursing ladder system [28]. Evidence suggests that advanced nurses (N3 and N4) have a better awareness of, beliefs in, attitudes toward, knowledge of, skills in, and behaviors of evidence-based practice than do new nurses (N, N1, and N2) [29]. Chi-square tests, Fisher’s exact tests, and t-tests were also conducted to compare the frequency of false alarms and response time to alarms. All data analyses were performed using the statistical software package Stata Statistical Software: Release 16 (College Station, TX: StataCorp LLC) [30]. A nominal significance level of 0.05 and power of 80% were used throughout the analysis.

## Results

**Fig. 2:**
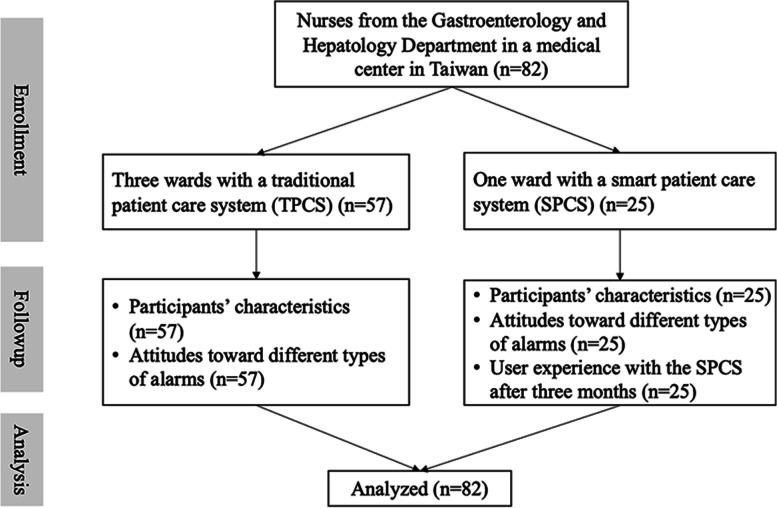
Flow diagram of participants

Eighty-two nurses from all four wards of the Gastroenterology and Hepatology Department were recruited and analyzed in this study (Fig. 2). Fifty-seven nurses were from the TPCS wards, and 25 nurses were from the SPCS ward. The characteristics of the participants are presented in Table 1. The average age was approximately 30 years old, and 85% of participants were female. Their average working experience in nursing was 6.8 years in total and 3.7 years in their current department. A total of 64.6% of the nurses were contract nurses, and 19.5% of them were registered nurses. Approximately 98.8% of the nurses had received a bachelor’s degree or above. There are slight differences in the characteristics between nurses from the TPCS and SPCS wards, which, however, are without statistical significance.

**Table 1:** Participant characteristics

	Total (*N* = 82)	TPCS (*N* = 57)	SPCS (*N* = 25)	*P-*value
Age (mean)	29.76 years	30.32	28.48	.349
Total working experience as a nurse (mean)	6.8 years	7.40	5.56	.347
Working experience in current department (mean)	3.7 years	3.91	3.36	.680
Sex				
Male	12 (14.6%)	7 (12.3%)	5 (20.0%)	.363
Female	70 (85.4%)	50 (87.7%)	20 (80.0%)	
Education level				.436
Associate’s degree	1 (1.2%)	0 (0.0%)	1 (4.0%)	
Postgraduate 2 years (PGY2)	18 (22.0%)	14 (24.6%)	4 (16.0%)	
Bachelor’s degree	57 (69.5%)	39 (68.4%)	18 (72.0%)	
Master’s degree	6 (7.3%)	4 (7.0%)	2 (8.0%)	
Job titles				
Administrative assistant	1 (1.2%)	0 (0.0%)	1 (4.0%)	.748
Contract nurse	53 (64.6%)	36 (63.2%)	17 (68.0%)	
Registered nurse	16 (19.5%)	12 (21.1%)	4 (16.0%)	
Practical nurse	6 (7.3%)	4 (7.0%)	2 (8.0%)	
Assistant head nurse	4 (4.9%)	3 (5.3%)	1 (4.0%)	
Head nurse	2 (2.5%)	2 (3.5%)	0 (0.0%)	
Clinical nursing ladder				
N	20 (24.4%)	15 (26.3%)	5 (20.0%)	.257
N1	29 (35.4%)	18 (31.6%)	11 (44.0%)	
N2	19 (23.2%)	16 (28.1%)	3 (12.0%)	
N3	7 (8.5%)	3 (5.3%)	4 (16.0%)	
N4	7 (8.5%)	5 (8.8%)	2 (8.0%)	

Nurses’ attitudes toward different alarms are presented in Table 2. The most annoying alarm was stated as being the emergency call alarm in both the TPCS and SPCS wards, with an average of approximately 60% of nurses agreeing to this statement, and 33% of them considered the bed-exit alert as the second most annoying alarm. For the most cumbersome alarm, over two-thirds of nurses in the TPCS wards considered the emergency call alarm the most cumbersome alarm, while the most cumbersome alarm perceived by nurses from the SPCS ward was the bed-exit alert (44%). In addition, less than 2% of nurses from the TPCS wards considered none of the alarms cumbersome, while 16% of nurses from the SPCS ward held such thoughts. These differences were statistically significant.

**Table 2:** Nurses’ attitudes toward different patient care system alarms (N = 82)

	Total N (%)	TPCS (*n* = 57)	SPCS (*n* = 25)	*P*-value
Most annoying alarm				
Normal call	6 (7.3%)	4 (7.0%)	2 (8.0%)	.868
Emergency call	49 (59.8%)	33 (57.9%)	16 (64.0%)	
Bed-exit alert	27 (32.9%)	20 (35.1%)	7 (28.0%)	
Desktop phone call	0 (0.0%)	0 (0.0%)	0 (0.0%)	
None	0 (0.0%)	0 (0.0%)	0 (0.0%)	
Most cumbersome alarm				
Normal call	6 (7.3%)	4 (7.0%)	2 (8.0%)	.005
Emergency call	47 (57.3%)	39 (68.4%)	8 (32.0%)	
Bed-exit alert	23 (28.1%)	12 (21.1%)	11 (44.0%)	
Desktop phone call	1 (1.2%)	1 (1.8%)	0 (0.0%)	
None	5 (6.1%)	1 (1.8%)	4 (16.0%)	
Most time-consuming alarm				
Normal call	12 (14.6%)	7 (12.3%)	5 (20.0%)	.003
Emergency call	34 (41.5%)	29 (50.1%)	5 (20.0%)	
Bed-exit alert	26 (31.7%)	18 (31.6%)	8 (32.0%)	
Desktop phone call	2 (2.4%)	1 (1.8%)	1 (4.0%)	
None	8 (9.8%)	2 (3.5%)	6 (24.0%)	
Most easily ignorable alarm				
Normal call	10 (12.2%)	8 (14.0%)	2 (8.0%)	.024
Emergency call	3 (3.6%)	2 (3.5%)	1 (4.0%)	
Bed-exit alert	40 (48.8%)	32 (56.1%)	8 (32.0%)	
Desktop phone call	9 (11.0%)	7 (12.3%)	2 (8.0%)	
None	20 (24.4%)	8 (14.0%)	12 (48.0%)	

Similarly, half of the nurses from the TPCS wards considered the emergency call alarm to be the most time-consuming alarm to which to respond, while it was the bed-exit alert that nurses from the SPCS ward rated as the most time-consuming alarm. In total, approximately one-fourth of nurses from the SPCS ward did not consider any alarm as being time-consuming to which to respond, while this percentage was only 3.5% in the TPCS group. These differences are also statistically significant. There is a large difference regarding the attitudes toward the most easily ignorable alarm in the TPCS and SPCS groups. Fifty-six percent of nurses from the TPCS wards considered the bed-exit alert to be the most easily ignorable alarm, and this rate was reduced to 32% in the SPCS ward. Furthermore, almost half of nurses (48%) considered no specific alarm to be easily ignorable with all the functions in the SPCS ward, while this proportion was only 14% in the TPCS wards.

Among the nurses in the SPCS ward (*n* = 25), we further investigated their user experience of this system with a 5-point Likert scale (Table 3). The agreement level was scored from 1 (strongly disagree) to 5 (strongly agree). The most helpful function of the SPCS was immediate communication via phone when compared with the TPCS, with a weighted average score of 3.92/5, and 52% of nurses strongly agreed that this function was helpful. The second highly ranked function was the three-stage bed-exit alert, with an average score of 3.68/5, and approximately 24% of nurses strongly agreed that this function was helpful. Regarding other specific functions of the SPCS, 52% of nurses agreed that the awareness of patients’ in-time situations was increased, 56% agreed that the event presentation was easy to understand, and 44% agreed that the interface was easy to use. In general, nurses found that care efficiency and quality were improved, the SPCS was better than the TPCS, and the stress caused by patient care was reduced.

**Table 3:** Nurses’ experience using the SPCS (*N* = 25)

	Agreement level					
	Strongly disagree	Disagree	Neutral	Agree	Strongly agree	Weighted average
The immediate communication via phone is helpful	1 (4%)	3 (12%)	6 (24%)	2 (8%)	13 (52%)	3.92
The three-stage bed-exit alert is helpful	0 (0%)	2 (8%)	10 (40%)	7 (28%)	6 (24%)	3.68
Care efficiency is improved	0 (0%)	1 (4%)	8 (32%)	16 (64%)	0 (0%)	3.60
The awareness of patients’ in-time situation is increased	0 (0%)	0 (0%)	12 (48%)	13 (52%)	0 (0%)	3.52
Care quality is increased	0 (0%)	1 (4%)	12 (48%)	12 (48%)	0 (0%)	3.44
The event presentation is easy to understand	0 (0%)	3 (12%)	8 (32%)	14 (56%)	0 (0%)	3.44
The interface is easy to use	0 (0%)	0 (0%)	14 (56%)	11 (44%)	0 (0%)	3.44
The stress caused by caring for patients is reduced	1 (4%)	2 (8%)	15 (60%)	7 (28%)	0 (0%)	3.12

A total of 230 alarms from patient pressing the call buttons or the automatic bed-exit alerts were observed with 120 alarms from the TPCS wards and 110 alarms from the SPCS ward. The average response time in TPCS was 145.66 s while it was 59.02 s in the SPCS (*P* < .001). In other words, the average response time was reduced by 59.48% in the SPCS ward in comparison with that in the TPCS wards. The impact of SPCS on the difference in the response time, however, should be interpreted with caution as other factors may influence the result. Among the 110 alarms in the SPCS ward, none of them were false bed-exit alarms. In comparison, among 120 alarms in the TPCS wards, 42 of them were false bed-exit alarms. The false alarm rate in SPCS and TPCS was 0, and 35%, respectively, with statistical significance (*P* < .001). In addition to the specification of alarm sources, SPCS allowed nurses to receive the signal by their smartphone. In this study, we found that 30.91% of alarms in SPCS were processed because nurses received the information by their phone.

## Discussion

This study examined nurses’ attitudes toward different alarms as well as their user experience of a smart patient care system. Emergency calls are treated as the most annoying alarm in both the TPCS and SPCS wards and as the most cumbersome and time-consuming alarm in the TPCS wards. Bed-exit alerts were perceived as the most cumbersome and time-consuming alarm in the SPCS ward. The SPCS successfully attracted the attention of nurses to bed-exit alerts due to its advantages, including the specification of alarm purposes, immediate communication with patients, and the three-stage bed-exit alert leading to the actual action of responding to it. Although the bed-exit alert was treated as the most cumbersome and time-consuming alarm, it was not considered the most annoying alarm, which may be because nurses want to be notified by this kind of alarm so that they can take active action to assist patients at high risk of falling. The most easily ignorable alarm was the bed-exit alert (56%) in the TPCS wards, while it was reduced to 32% in the SPCS ward. In addition, 48% of nurses claimed that none of the alarms were easily ignorable with the SPCS. Regarding the user experience of the SPCS, the most helpful function of the SPCS was considered to be immediate communication via phone, followed by the three-stage bed-exit alert. The advantages of the SPCS over the TPCS perceived by nurses include an improvement of care efficiency, an increase in the awareness of patients’ in-time situation and care quality, an easily understandable event presentation, an easy-to-use interface, and a reduction in stress caused by caring for patients.

Patient care system alarms, by nature, are a source of interruption for nurses [4, 21, 24]. These interruptions have been shown as a potential contributory factor leading to medical errors in clinical environments [31–33]. Many studies have tried to reduce medical errors by reducing the frequency of interruptions, the effect and safety of which, however, remain questionable [21, 34, 35]. The main reason for this may be due to the complex nature of interruptions in hospital settings, in which not all interruptions are undesirable [14, 17, 20, 24]. Moreover, patients think that it is essential that their requests are taken seriously and that they can receive support whenever needed [2, 7]. Studies have shown that a patient care system helps patients feel safe and contributes to their successful recovery [2, 3]. Interruptions such as alarms also play an important role in transferring requests and information in a timely manner and enhancing patient care quality [5, 14, 22, 36].

Nurses have a variety of clinical and administrative tasks and have to decide whether to prioritize responding to an alarm over the ongoing activity [37]. There are different levels of importance or urgency in which nurses find themselves when receiving an alarm [24]. Before actually responding to an alarm, nurses need to assess the situation, figure out how to respond, and decide what to do [26]. Therefore, a resilient patient care system is necessary to help nurses adjust their work to various work conditions [38]. One of the critical functions that a resilient patient care system should have is the capacity through which nurses can access an alarm immediately wherever and whenever needed. In the TPCS wards in our study, if patients press the nurse call button or leave their bed, thus setting off the bed-exit alert, nurses can only receive information on the bed number of the alert from a fixed display such as the patient’s room door light and display monitors in the corridor or nursing station. Thus, it is highly likely that nurses will not see this fixed display if they are not located near it.

Studies have shown that nurses tend to perceive alarms as interruptions rather than means through which to communicate with patients [16]. In the SPCS ward of our study, all alarms are routed to nurses’ smartphones, and they are supposed to carry their phones around to receive any nurse alarm whenever and wherever necessary. This smartphone integration in the SPCS is able to notify highly mobile nurses when they are not located near a fixed display [23]. The results from the present study showed a significant reduction in the rate of the most easily ignorable alarm perceived by nurses from 86% in the TPCS wards to 52% in the SPCS ward. On the other hand, there were still half of the nurses who thought the SPCS alarms could be ignored somehow. Unfortunately, we failed to collect data on reasons that could lead to this result which should be further investigated in the future. The incorporation with nurses’ smartphones in the SPCS helps reduce the limitation of location when receiving alarms and therefore, may increase the frequency of using the patient care system. Evidence has shown that a higher frequency of patient care system usage is associated with a lower hospital fall incidence, higher patient satisfaction, and increased intention to use the patient care system [39].

Moreover, an SPCS may also raise the nurse interruptions and their work burden. As a resilient system, the frequency of interruptions does not necessarily increase in the SPCS ward and may even decrease by engaging with the nurse scheduling system data. This function allows nurses to only receive alarms from patients who have been primarily assigned to them during their shift. Studies have shown that nurses are more likely to prioritize an alarm if they have primary responsibility for the patient who is calling [14, 36]. Therefore, routing alarms to primarily assigned nurses by integration with the nursing scheduling system helps nurses receive fewer but more relevant alarms, which is better for their decision-making process in terms of the prioritization of responding to alarms. In addition, the SPCS is able to transfer a signal to the nursing station if no one responds after 20 s to ensure that the alarm is received and addressed. In practical situations, nurses will also choose to pass their work phone to another colleague before they undertake a scheduled task requiring high concentration that cannot be disrupted. This approach is practical yet not the best solution. It would be more convenient if the patient care system were to allow nurses to formally hand over their responsibility to secondarily assigned nurses in the system just by clicking his/her name in the app when necessary. This function was not integrated into the SPCS during the study period, and thus, it is suggested as a direction for future research. Studies have consistently shown that formal responsibility handover helps nurses focus on ongoing tasks [14, 24, 36].

It is suggested that a patient care system capable of showing the purpose of an alarm helps nurses save time struggling and improve patient care accordingly [4, 7, 21]. Such a patient care system should be able to display an alarm as a normal call, emergency call, or any other alarms needed in nursing practices. Currently, few patient care systems in hospitals have such functions, and limited evidence has been found in the existing scientific literature [6, 40]. Studies have shown that an alarm is easier to ignore, especially when the purpose of the alarm is not specified [22, 24]. There are four major purposes of alarms at our study site: a normal call, an emergency call, a bed-exit alert, and a desktop phone call. The SPCS can display each alarm’s purpose on monitors or phones, while nurses in the TPCS wards were not able to differentiate between normal calls and bed-exit alerts. Given the higher frequency of normal calls, it is easier for nurses in the TPCS wards to ignore bed-exit alerts compared to nurses in the SPCS ward. The rate of nurses’ perception of missing a bed-exit alert is approximately 56% in the TPCS wards, while this rate was reduced to 32% in the SPCS ward.

The results from studies investigating the purposes of alarms show that only a small number of such alarms require immediate attention and handling by nurses [4, 7, 21]. Moreover, nurses are suggested to prioritize responding to alarms to improve patient-centered care quality and reduce adverse events [6, 7]. Instead of focusing on fostering nurses’ attitudes toward alarms, which has been shown to have an unoptimistic effect [41], more effort should be made to develop resilient technologies that can support nurses in better prioritizing multiple patient care duties [6, 42]. The concept of resilience engineering is also applied in the SPCS introduced at our study site with specifying alarm purposes and allowing for immediate communication with patients directly via phone. Normally it is difficult for nurses to decide whether the alarm is so urgent that they should abort the ongoing task based on limited information. In the SPCS ward, nurses can directly communicate with a patient via phone for more details on the purpose of the alarm and may even ask him/her to wait for a short period for their assistance if the situation allows. The results from our study showed that over half of nurses agreed that their awareness of patients’ in-time situations was increased via this system. In addition, the statement “immediate communication via phone is helpful” has the highest agreement level among all the innovative functions of the SPCS. The function of immediate communication via phone in SPCS helps nurses respond to patients’ requests or needs in a timely manner and to have a better capacity to prioritize multiple clinical tasks.

Patient safety is an important aspect of a patient care system, from the perspective of both nurses [6] and patients [7]. Patient falls are serious patient safety problems in hospitals, and fall prevention is one of the most important expectations of the patient care system [11, 43]. Hospital falls commonly occur between 5 pm and 7 am in a patient’s room when staffing levels are lower, and a large number of falls are related to getting in and out of bed [13]. In clinical practice, it is common to use bed-exit detection systems, such as mattresses/pads, cameras, and wearable devices, to help notify nurses about bed-exit situations for patients who are at high risk of falling [44, 45]. The effect of these systems on fall prevention, however, is inconclusive [46, 47]. One of the major concerns of the bed-exit system is that there is not enough time for nurses to receive an alert and to then provide actual assistance before the patient leaves his/her bed [25, 48]. In our study, the SPCS incorporated a motion-sensing mattress that used a machine learning algorithm that allowed for three-stage bed-exit notification so that nurses could receive the alert about the patient’s intention to leave his/her bed as early as when he/she sits up in bed. In addition, nurses could ask patients to wait for a short period for assistance in leaving bed with the immediate communication function of the SPCS when they found themselves unable to provide assistance immediately. With the three-stage bed-exit notification and immediate communication functions, the SPCS can help nurses respond to and assist patients at risk of falling when they intend to leave bed in a timely manner. There is also a difference in the administrational response process between the SPCS and TPCS wards. In the TPCS wards, once the bed-exit alert is activated, nurses can simply stop it by pressing a button at the nursing station, while in the SPCS ward, they are required to stop it at the patient’s bedside. Evidence suggests that responding to a situation implies dealing with the actual situation, especially when the outcome is related to patient safety [24, 38]. Although the response process in the SPCS ward may bring about some inconvenience for nurses, it helps ensure that patients’ needs are actually being addressed. Therefore, we observed that although the rate of nurses perceiving bed-exit alerts as the most cumbersome type of alarm was higher in the SPCS ward than in the TPCS wards, the proportion of them considering such alerts as the most annoying alarm decreased. In addition, over half of the nurses in the present study agreed that the bed-exit alert was helpful.

False alarms have been commonly reported in the existing bed-exit alarm systems. It is shown that false alarms have counted for above 80% of all bed-exit alarms leading to alarm fatigue which can increase the nursing staff’s work burden and lower their willingness to use the system and finally result in falling [49–51]. The excessive false alarms may lead the alarm fatigue in which nursing staff would occasionally ignore the alarms. A multicenter study showed that over 70% of the monitor alarms were false alarms and only 5.9% of all alarms received responses from nursing staff [52]. In addition, false alarms may frequently disrupt patients’ sleep and negatively influence their recovery [53]. In this study, we observed 35.2% of false alarms in TPCS and 0% in SPCS. The high predictive positive value of SPCS significantly improves nursing staff’s willingness to use the system which may be another reason that SPCS has a positive effect on fall prevention [54, 55].

This study has some limitations. First, the patient care requirements and nurse-patient interactions vary among different departments in hospitals [36]. In our study, the smart patient care system was only introduced in one ward, limiting the study in its generalizability to different settings. Second, the small sample size limits the generalizability of this study. We collected data on 82 nurses’ attitudes toward patient care systems were collected, while only 25 nurses used the SPCS and gave us feedback on their user experience. This sample size may be relatively small compared with those of other studies. There are no consistent recommendations for sample size in usability studies, with some suggesting that five is sufficient, and others suggesting that there should be ten or more [56]. Third, a patient care system involves both nurses and patients [2, 22]. However, we only focused on the perspectives and experiences of nurses. Future work should also collect data from patients and their family members. Lastly, many factors can affect nurses’ response time such as ward architecture, route length, conditions of nurses [36, 54]. These potential influencing factors were not taken into analysis in the present study which could cause bias for the impact of the SPCS on the attitude, experience, and response time of nurses.

## Conclusions

Nurses are required to handle multiple tasks in clinical practice, and they tend to consider patient care system alarms as being annoying, cumbersome, time-consuming, and easily ignorable. Conversely, alarms play an important role in the transfer of patient requests, and responding to them in a timely manner is associated with reduced injuries and increased patient satisfaction [39]. A resilient patient care system is needed to help nurses prioritize their responses to alarms and cope with them while also handling other nursing duties. These patient care systems should be able to specify alarm purposes, such as normal, emergency, and bed-exit alarms, to help nurses quickly judge the level of urgency; route the alarm directly to the mobile phone/device carried by the primary assigned nurse of the calling patient to reduce the frequency of alarms and increase the response rate; be capable of immediate communication to help nurses obtain more details of the calling purpose or even buy some more time to provide assistance; and include in-advance bed-exit alerts that provide nurses with sufficient time to provide assistance. Future product development for patient care systems should focus on the capacity that allows nurses to make more informed prioritization decisions between responding to alarms and the ongoing tasks at hand and finally assist them in adjusting their work in various situations to improve work efficiency and care quality. Further research is also needed on the effect of patient care systems on nurses’ work efficiency and patient safety outcomes, such as fall prevention.

## Data Availability

The dataset used during the current study is available from the corresponding author on reasonable request.

## Abbreviations

SPCS: Smart patient care system
TPCS: Traditional patient care system
